# Major changes in chromosomal somy, gene expression and gene dosage driven by Sb^III^ in *Leishmania braziliensis* and *Leishmania panamensis*

**DOI:** 10.1038/s41598-019-45538-9

**Published:** 2019-07-01

**Authors:** Luz H. Patino, Hideo Imamura, Lissa Cruz-Saavedra, Paula Pavia, Carlos Muskus, Claudia Méndez, Jean Claude Dujardin, Juan David Ramírez

**Affiliations:** 1 Grupo de Investigaciones Microbiológicas-UR (GIMUR), Programa de Biología, Facultad de Ciencias Naturales y Matemáticas, Universidad del Rosario, Bogotá, Colombia; 2 Molecular Parasitology Unit, Department of Biomedical Sciences, Institute of Tropical Medicine, Antwerp, Belgium; 3 Unidad de Investigación Científica, Subdirección de Docencia e Investigación, Hospital Militar Central, Bogotá, Colombia; 4 Programa de Estudio y Control de Enfermedades Tropicales (PECET), Facultad de Medicina, Universidad de Antioquia, Medellín, Colombia; 5 Dirección de Sanidad Militar, Ejercito Nacional de Colombia, Bogotá, Colombia; 6 Department of Biomedical Sciences, University of Antwerp, Antwerp, Belgium

**Keywords:** Parasite genomics, Clinical microbiology

## Abstract

*Leishmania braziliensis* and *Leishmania panamensis* are two species clinically and epidemiologically important, among others because of their relative resistance to first-line drugs (antimonials). The precise mechanism underlying the ability of these species to survive antimony treatment remains unknown. Therefore, elucidating the pathways mediating drug resistance is essential. We herein experimentally selected resistance to trivalent antimony (Sb^III^) in the reference strains of *L*. *braziliensis* (MHOM/BR75/M2904) and *L*. *panamensis* (MHOM/COL/81L13) and compared whole genome and transcriptome alterations in the culture promastigote stage. The results allowed us to identify differences in somy, copy number variations in some genes related to antimony resistance and large-scale copy number variations (deletions and duplications) in chromosomes with no somy changes. We found mainly in *L*. *braziliensis*, a direct relation between the chromosomal/local copy number variation and the gene expression. We identified differentially expressed genes in the resistant lines that are involved in antimony resistance, virulence, and vital biological processes in parasites. The results of this study may be useful for characterizing the genetic mechanisms of these *Leishmania* species under antimonial pressure, and for clarifying why the parasites are resistant to first-line drug treatments.

## Introduction

Leishmaniases are a group of parasitic diseases caused by Protozoa belonging to the genus *Leishmania*^1,2^. These diseases are characterized by a broad spectrum of clinical manifestations, including cutaneous leishmaniasis (CL), mucosal leishmaniasis (ML), and visceral leishmaniasis (VL)^3^. Being endemic in 87 countries, CL is the most common form of leishmaniasis with an estimated of 0.6–1.0 million cases annually^4^. These clinical manifestations have been associated with diverse *Leishmania* species, including Old World species such as *L*. *major*, *L*. *tropica*, *L*. *aethiopica* and *L*. *infantum*^5,6^; and New World species such as, *L*. *braziliensis*, *L*. *amazonensis L*. *mexicana*, *L*. *panamensis* and *L*. *guyanensis*^7–10^.

*L*. *braziliensis* and *L*. *panamensis* are very important in clinical and epidemiological terms, not only because these species are widely distributed in Latin America^7,11^ but also because infections by these species, especially *L*. *braziliensis*, have a substantially greater potential to manifest as ML^12,13^, and for the relatively unresponsiveness to first-line drugs [e.g., antimonials like sodium stibogluconate (SSG)]. Previous studies revealed that in some Latin American countries (e.g., Brazil, Peru, Guatemala, and Colombia), the therapeutic failure rates are between 25–40% for patients with CL caused by *L*. *braziliensis*^14–16^ and 15–31% for patients with CL caused by *L*. *panamensis*^10,17–19^. However, numerous factors influence the final therapeutic outcome of antimonial treatments, related to the host, the drug and the parasite^20,21^. Moreover, the biological features of parasites might play an important role in this process^21–24^.

To date, various approaches have been applied, including next generation sequencing techniques (genomics, transcriptomics), proteomic and metabolomic analysis, to characterize and establish the relationship between some *Leishmania* species and the mechanisms underlying antimony resistance^25–39^. These studies have demonstrated that under drug pressure and in the absence of transcriptional regulation, *Leishmania* uses several adaptive mechanisms to modulate the gene dosage of therapeutic targets or other determinants of resistance, such as the generation of episomal amplicons^34^, aneuploidy and/or local gene copy number variation (CNV)^32,33,40^, and single-nucleotide polymorphisms (SNPs) in drug targets or transporters^35^.

These adaptive mechanisms have been mainly studied in Old world species, such as *L*. *donovani*^35,41^, *L*. *tropica*^42^ and *L*. *infantum*^27,28^. Comparative genomic and transcriptomic analysis (DNA-seq and RNA-seq) of lines sensitive and resistant to trivalent antimony (Sb^III^) have revealed that these species alter the copy number of particular genes either by a local copy number variation or by modulating a copy number of the whole chromosome^25,42^. These copy number variations affected specific genes like genes encoding transport proteins, such as ABC transporter MRPA^34,42^, aquaglyceroporin 1 (AQP1)^28,35^; genes encoding proteins essential for virulence, such as amastin and GP63^37,38^; or genes encoding proteins associated with the trypanothione biosynthesis pathway, such as gamma-glutamylcysteine synthetase^38^.

Despite the advances that have enabled to characterize the mechanisms underlying the drug resistance of *Leishmania* species, the genome and transcriptome level changes occurring in New World *Leishmania* parasitic species (mainly in the subgenus *Viannia*) in response to stresses (e.g., drug treatments) remain not well characterized. Therefore, in this study, we performed comparative genomic and transcriptomic analyses of the experimentally selected Sb^III^ resistant strain and wild type sensitive strain of two of the main clinically and epidemiologically important *Leishmania* species in Latin America, namely *L*. *braziliensis* and *L*. *panamensis*.

## Results

### Induction of Sb^III^ resistance in *L*. *braziliensis* and *L*. *panamensis* lines

Initially, we selected *in vitro* populations of *L*. *braziliensis* and *L*. *panamensis* that were resistant to Sb^III^. The selection process was initiated in quadruplicates, starting with 1.5 and 1.8 µg/mL Sb^III^ for *L*. *braziliensis* and *L*. *panamensis* respectively, with six rounds of selection to each species as described in materials and methods. The selection dynamics for both species was similar; for *L*. *braziliensis*, two replicates did not survive the second (3 µg/mL Sb^III^) and fourth (3 µg/mL Sb^III^) rounds of selection, respectively and two replicates were successfully selected to survive the highest Sb^III^ concentration (48 μg/mL). In *L*. *panamensis* the behavior was similar, two replicates did not survive the third (7.2 µg/mL Sb^III^) and fifth (28.8 µg/mL Sb^III^) rounds of selection, respectively and two replicates were successfully selected to survive the highest Sb^III^ concentration (57.6 μg/mL). When we evaluated the time of resistance, which was defined as the time needed for each line to display a similar growth curve compared to the parental line in the presence of 48 μg/mL Sb^III^ for *L*. *braziliensis*, and 57,6 μg/mL Sb^III^ for *L*. *panamensis*. We observed that the time estimated of resistance for *L*. *braziliensis* was 14 weeks and for *L*. *panamensis* was 18 weeks. Finally, we evaluated the stability of the resistance phenotype, in which all the Sb^III^-resistant lines were maintained for 4 weeks in medium without Sb^III^. The results obtained demonstrated that the index of resistance of each line remained, suggesting that the *in vitro* selected drug resistance phenotype was stable.

### Chromosome/gene copy number variations

#### Chromosome copy number variations

**Figure 1:**
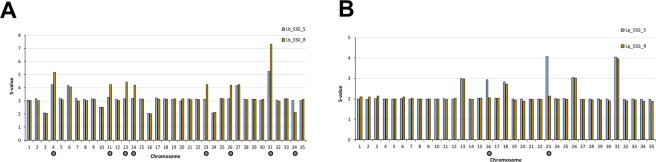
Dynamics of ploidy in *L*. *braziliensis* and *L*. *panamensis*. Comparisons of the chromosomal copy number between lines sensitive and resistant to Sb^III^ in *L*. *braziliensis* (left) and *L*. *panamensis* (right). The grey points indicate the chromosomes that underwent a change in somy.

**Figure 2:**
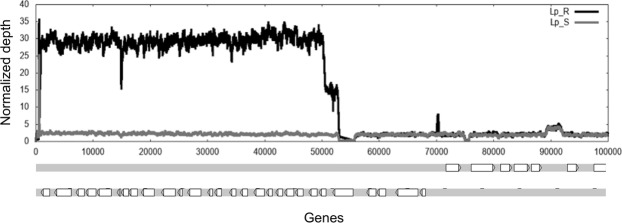
Intrachromosomal duplication in Sb^III^-resistant *L*. *panamensis* mutants. Raw read depth for chromosome 27 of the Sb^III^-resistant and -sensitive lines. The black and gray lines show the raw depth in Lp_SSG_S and Lp_SSG_R lines, respectively. The figure below is a representation of genes located in this region.

For each chromosome, the median somy value S within each sample, its corresponding median absolute deviation across reads of that chromosome, and the statistical significance of differences in S values between samples were calculated as described in materials and methods. When we compared the somy levels in the SSG_R and SSG_S lines, we observed that the S values of eight chromosomes in the Lb_SSG_R line changed regarding to Lb_SSG_S line. In seven chromosomes, the S values increased significantly: chromosomes 11, 13, 14, 23, and 26 (trisomic to tetrasomic), chromosome 4 (tetrasomic to pentasomic), and chromosome 31 (pentasomic to heptasomic); and in one chromosome: chromosomes 34 the S values decreased (trisomic to disomic), the rest of the karyotype remained unchanged (trisomic) (Fig. 1A). In the Lp_SSG_R line, we observed that the S values of chromosomes 16 and 23 decreased significantly: chromosome 16 (trisomic to disomic) and chromosome 23 (tetrasomic to disomic), and the rest of the karyotype remained unchanged (disomic) (Fig. 1B). Additionally, a uniform increase in read depth was observed for the first 50,000 bp of chromosome 27 in the Lp_SSG_R line (Fig. 2). This concerned 23 genes that were amplified between 8–19 times more regarding to Lp_SSG_S line (Supplementary Table S1).

The S-Values of *L*. *braziliensis* and *L*. *panamensis* were consistent with the somy values predicted based on the alternative allele frequency profile. The allele frequency counts for each predicted heterozygous SNP did not exhibit a discordance between read depth and allele frequencies, confirming the accuracy of the previously described somy profiles.

#### Gene copy number variation (CNV)

**Figure 3:**
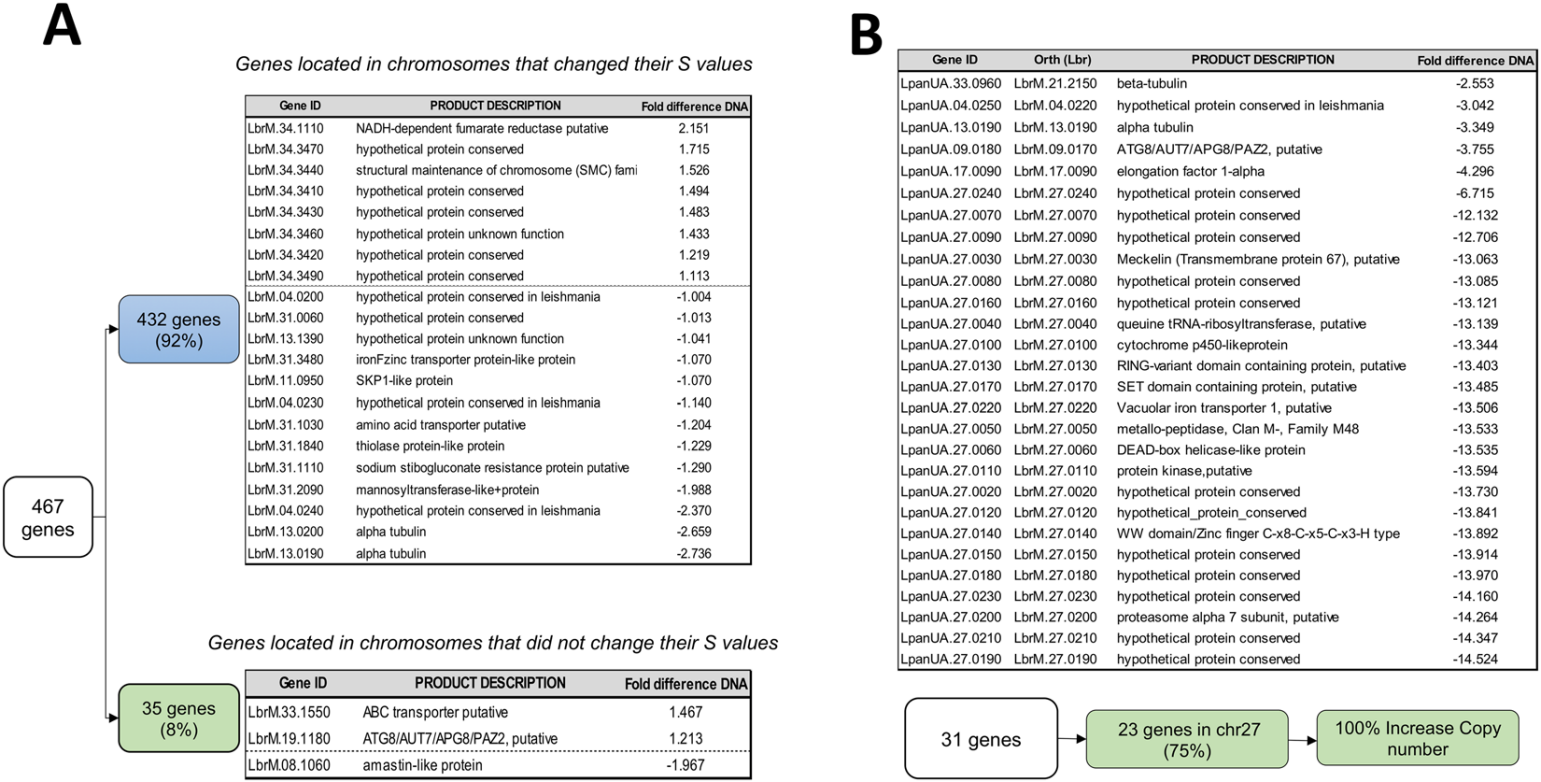
Gene copy number variation between sensitive and resistant lines of *L*. *braziliensis* and *L*. *panamensis*. Selection of genes with different copy numbers in the SSG_S and SSG_R lines (difference >1.0) in (**A**) *L*. *braziliensis* and (**B**) *L*. *panamensis*. The genes in *L*. *panamensis* were listed along with their corresponding *L*. *braziliensis* ortholog genes.

We examined the gene CNVs between the SSG_S and SSG_R lines and found that in *L*. *braziliensis*, the changes in the chromosome copy number matched the gene copy numbers for the same chromosomes, as expected. Specifically, a total of 467 genes presented CNV between the Lb_SSG_S and Lb_SSG_R lines (Z score >2, equivalent to p < 0.05). Of these genes, 432 (92%) were located on eight chromosomes whose somy had changed, whereas the remaining 35 genes (8%) were located on chromosomes whose somy remained the same (Supplementary Table S2). After applying a stricter difference cut off (gene depth difference between Lb_SSG_R and Lb_SSG_S greater than 1.0), we detected in the chromosomes with change in the somy, 13 genes with higher copy number and 8 genes with lower copy number in the Lb_SSG_R than in the Lb_SSG_S line (Fig. 3A), likewise, 3 genes with variation in the copy number between both lines in the chromosomes without change in the somy (Fig. 3A).

Most of the genes with a lower copy number in the Lb_SSG_R line were annotated as hypothetical proteins. Of the genes with a higher copy number in the Lb_SSG_R line, the following five genes appeared functionally intriguing: two genes encoding an alpha-tubulin, with three extra copies (LbrM.13.0190 and LbrM.13.0200), two genes encoding transporter proteins, with one extra copy (amino acid transporter: LbrM.31.1030 and iron-zinc transporter: LbrM.31.3480), and one gene encoding a protein associated with antimony resistance, with one extra copy (sodium stibogluconate resistance protein: LbrM.31.1110) (Fig. 3A).

In contrast, we detected fewer differences in the gene copy numbers per chromosome between the Lp_SSG_S and Lp_SSG_R lines. We observed that 31 genes had different copy numbers between the two lines (Z score >2, equivalent to p < 0.05). Of these 31 genes, 23 (74%) were located on chromosome 27, most strikingly, the copy number for many of the genes was more than 10 times higher in the Sb^III^-resistant line than in the Sb^III^-sensitive line (Fig. 3B). As for the potential mechanism of this amplification, it spanned between the base position between 1 and 53055 bp of chromosome 27, ending in a protein kinase gene (LpanUA.27.0110/LbrM 27.0110) that contains a gap in the middle. We identified 472 bases, for which there were 4 direct and 6 reverse paralogs. These repeats are not a part of a known transposon, but they may be potentially involved in the formation of this duplication.

### Single nucleotide polymorphisms (SNPs)

**Figure 4:**
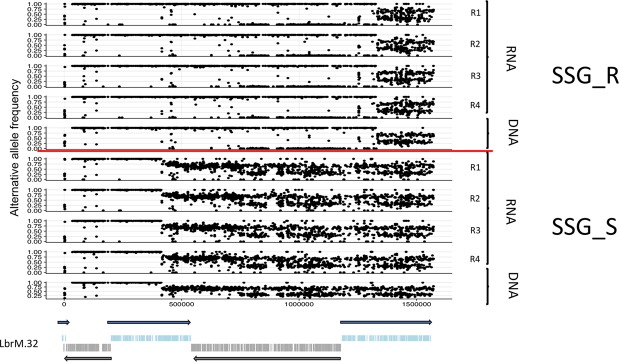
Single nucleotide polymorphism (SNPs) in the chromosome 32 of *L*. *braziliensis* sensitive and resistant to Sb^III^. The figure shows the SNPs (DNA/RNA) found in the chromosome 32, comparing the resistant and sensitive lines to Sb^III^. The figure below is a representation of the gene transcription in chr 32.

The comparison with the reference *L*. *braziliensis* sequence identified 35,878 SNPs in the Lb_SSG_S line and 35,689 SNPs in the Lb_SSG_R line. We evaluated the differences in the allele frequencies between the Lb_SSG_S and Lb_SSG_R lines and found that 176 heterozygous SNPs exhibited an allele frequency shift greater than 0.33 (Supplementary Table S3). Of these SNPs, remarkably 155 (88%) were located on chromosome 32, and the remaining 21 were located on other chromosomes. Of the SNPs on chromosome 32, we determined that 34 were present in the Lb_SSG_S line, but not in the Lb_SSG_R line (Fig. 4, Supplementary Table S3). Most of the SNPs found only in the Lb_SSG_S line were located in genes encoding hypothetical proteins, except one in a gene encoding an aquaporin-like protein (position 5642), leading to a change in the amino acid sequence (glutamine to glycine). Interestingly, this change was not observed in the Lb_SSG_R line (Table 1). Additionally, of SNPs on chromosome 32 detected in both Lb_SSG_S and Lb_SSG_R lines, four of them were located in genes encoding proteins associated with antimony resistance, one SNP in a gene encoding an ABC transporter protein (LbrM.32.2270) and the other three SNPs located in different positions of a gene encoding a mitogen-activated protein kinase (Table 1).

**Table 1:** List of mainly SNPs found in the chromosome 32.

Gene ID	a	b	c	d	Product description	Lb_SSG_S DNA	Lb_SSG_R DNA	Lb_SSG_S RNA	Lb_SSG_R RNA
LbrM.32.2610	5642	C/G	Glu-Gln	308	aquaporin-like protein	0.326	0.000	0.3838	0.0025
LbrM.32.3540	51288	A/C	Gln-Pro	800	mitogen-activated protein kinase, putative	0.691	1.000	0.6630	0.9988
LbrM.32.3540	44169	G/T	Ala-Ser	792	mitogen-activated protein kinase, putative	0.680	1.000	0.6495	0.9958
LbrM.32.3540	44274	A/G	Lys-Arg	914	mitogen-activated protein kinase, putative	0.639	1.000	0.6440	0.9950
LbrM.32.2270	28477	T/C	ILe-Met	387	ABC transporter-like protein	0.557	1.000	0.7053	1.0000

^a^SNP position on the gene, ^b^Change of nucleotide respect to reference (Ref/Alt), ^c^Change in the amino acid and ^d^ SNP position on the protein.

In contrast to *L*. *braziliensis*, we did not identify many SNPs in *L*. *panamensis* or no major differences in the allele frequency between the Lp_SSG_S and Lp_SSG_R lines. Notably, on the highly duplicated region of chromosome 27 of Lp_SSG_R line descibed above, a major shift in the allele frequency between the Lp_SSG_S and Lp_SSG_R lines were observed. Specifically, average allele frequency differences between the Lp_SSG_S and Lp_SSG_R lines were 0.38 ± 0.08 and 0.06 ± 0.07 on the 44 heterozygous SNPs on duplicated region and on the 214 heterozygous SNPs on the non-duplicated region, respectively, where a standard deviation was shown with ± . The significance of separation of these differences was *p* = *3*.*6* × *10*^−*96*^ measured by a two-sided t-test. Little or no indels were observed in both *L*. *braziliensis* and *L*. *panamensis*.

Finally, we constructed a neighbor-joining network to observe genetic distances based on the SNPs between the Sb^III^-sensitive and -resistant lines, including RNA and DNA. For both species (Supplementary Fig. S1), the results demonstrated a clear separation between the Sb^III^-sensitive and -resistant lines. The RNA samples showed higher base difference with respect to their corresponding DNA samples mainly due to their depth variation not transcriptional strand biases because these were not observed based on the alternative allele frequency data.

### Relationships among the somy, gene dosage (CNV), and gene expression levels

**Figure 5:**
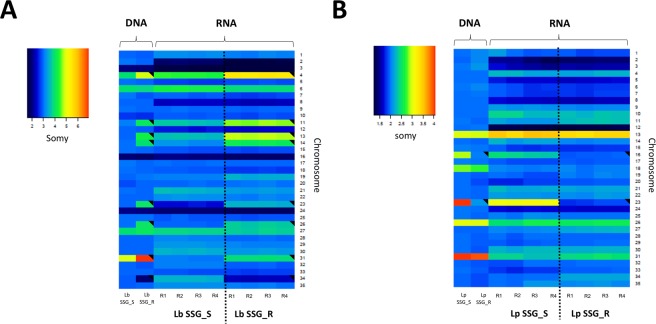
Relationship between chromosomal copy number variations and gene expression levels in the SSG_S and SSG_R lines of *L*. *braziliensis* and *L*. *panamensis*. The heatmaps show median normalized read depths of 35 chromosomes (y axis) found in *L*. *braziiensis* (Left) and *L*. *panamensis* (right) sensitive and resistant to Sb^III^ (x axis) The color key indicates the somy value (S), which ranged from 1 to 5 as follows: monosomy, S < 1.5; disomy, 1.5 ≤ S < 2.5; trisomy, 2.5 ≤ S < 3.5; tetrasomy, 3.5 ≤ S < 4.5; and pentasomy, 4.5 ≤ S < 5^40^. A black triangle in an upper right corner indicates a significant change in S value. R (replicates).

For each chromosome, we computed the median transcript level and compared this with DNA-based S values. The results obtained allow us to observe that in Lb_SSG_R and Lp_SSG_R lines, all chromosomes that presented changes in S values showed alterations of RNA in the same direction (Fig. 5A,B). This demonstrates that most of the gene expression changes were concordant with the chromosomal somy changes. Later, we investigated the dependency of gene expression on somy by regression analysis, between the relative chromosomal somy and the average relative gene expression levels of the SSG_R and SSG_S lines. Regarding the Lb_SSG_R *vs* Lb_SSG_S comparison, we detected a significant correlation, with a Pearson correlation R^2^ = 0.911 (*p* = 6.13 × 10^−19^) and a slope of 1.00. In contrast, for the Lp_SSG_R *vs* Lp_SSG_S comparison, the R^2^ and slope were 0.885 (*p* = 4.66 × 10^−17^) and 0.97, respectively.

We also evaluated the impact of the gene CNVs on gene expression levels. In *L*. *braziliensis*, we observed that of the genes with different copy numbers between the Lb_SSG_S and Lb_SSG_R lines (difference >1.0), 54% of them were differentially expressed in the two lines (greater than 1.5-fold difference). The genes exhibiting upregulated expression in the Lb_SSG_R line were mainly located on chromosomes 4, 8, 11, 13, and 31, whereas the downregulated genes were mostly on chromosomes 34 and only one in chromosome 33 (Supplementary Fig. S2).

Among upregulated genes in the Lb_SSG_R line we highlight three of them whose differential expression was associated with the gene CNV: LbrM.31.3480 (an iron-zinc transporter like-protein), LbrM.08.1060 (amastin-like protein), and LbrM.13.0190 (alpha-tubulin). Among the downregulated genes in the Lb_SSG_R line, two of them were LbrM.34.1110 (putative NADH-dependent fumarate reductase) and LbrM.33.1550 (putative ABC transporter) (Supplementary Fig. S2).

Even though Sb^III^ did not substantially affect the local gene/chromosome CNVs in *L*. *panamensis*, we observed that among the 31 genes with different copy numbers between the Lp_SSG_S and Lp_SSG_R lines, 28 (90%) were differentially expressed (by more than 1.5-fold). Thirteen of these genes encode hypothetical proteins without a known function. The other 15 genes encode known proteins and interestingly, all these genes exhibited upregulated expression in the Lp_SSG_R line. Specifically, LpanUA.33.0960/LbrM.21.2150 (beta tubulin), LpanUA.13.0190/LbrM.13.0190 (alpha-tubulin) and LpanUA.27.0140/LbrM.27.0140 (WWW domain/zinc finger C-x8-C-x5-C-x3-H-type protein). Finally, we observed the latter gene presented an expression level 100-fold higher in the Sb^III^-resistant line than in the Sb^III^-sensitive line (Supplementary Fig. S2).

Although the transcription levels were concordant with the somies and with local CNV in most of the chromosomes, we observed that the transcription of some specific genes do not follow this overall trend. A potential mismatch between transcriptional level and the local CNV was observed (Supplementary Fig. S3).

### Differentially expressed genes between SSG_S and SSG_R *L*. *braziliensis* and *L*. *panamensis* lines

**Figure 6:**
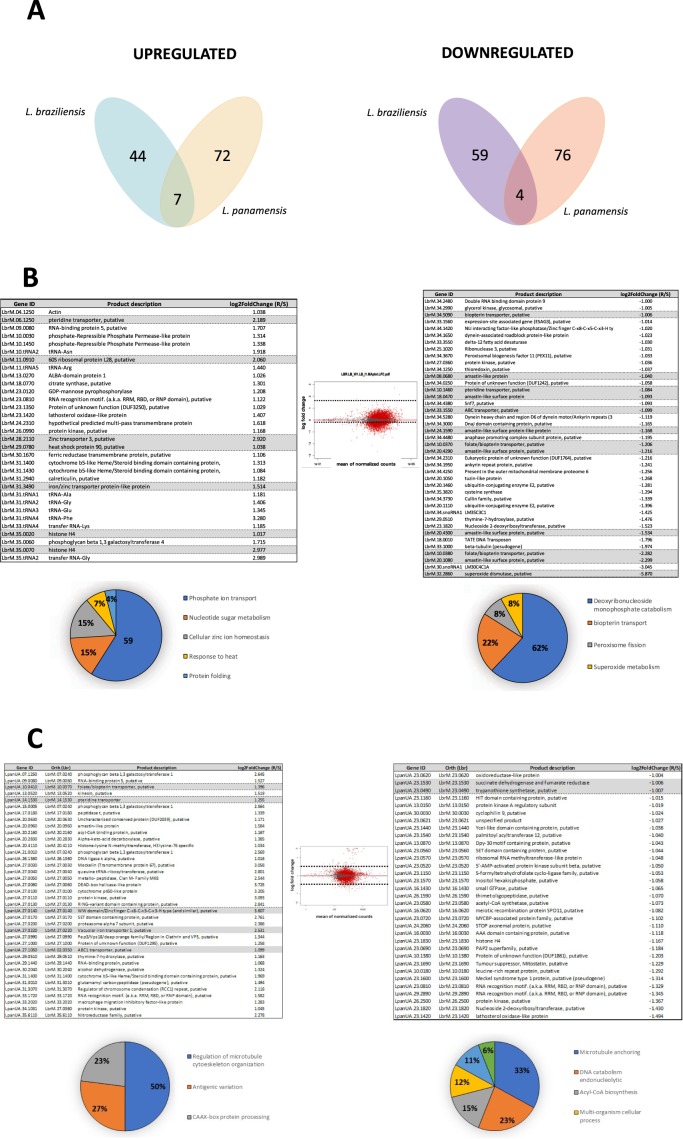
Transcriptional profile of the SSG_R and SSG_S lines in *L*. *braziliensis* and *L*. *panamensis*. (**A**) Venn diagram showing transcripts down- and up-regulated more than 2-fold in the resistant lines regarding the expression levels found in the parental sensitive line. The number of transcripts having significantly altered levels (up and down regulated) and the number of transcripts with DE when both species were compared (middle of each diagram) (**B**,**C**) Differentially expressed transcripts between the experimental conditions. B *(L*. *braziliensis*). C (*L*. *panamensis*). The graphic in the middle represents the MA plot constructed based on the DESeq2 results. Red points indicate significance at a 10% adjusted p-value, up-regulation and down-regulation respectively. Grey triangles indicate transcripts that showed no change. The lower figures present the results of the Gene Ontology enrichment analyses for biological process of up (left) and down (right) regulated transcripts. The transcripts included had an expression value higher than 2-fold change in both comparisons. The number of transcripts with a GO term is indicated in the corresponding pie slice.

The data for *L*. *braziliensis* revealed 844 differentially expressed genes between the SSG_S and SSG_R lines, among which 103 had a fold-change ≥2 in the Lb_SSG_R line, with 44 and 59 genes exhibiting upregulated and downregulated expression, respectively (Supplementary Table S4). In *L*. *panamensis*, 803 genes were differentially expressed, and 148 genes had a fold-change ≥2 in the Lp_SSG_R line, with 72 and 76 genes exhibiting upregulated and downregulated expression, respectively (Supplementary Table S5). A Venn diagram illustrated the number of significantly upregulated/downregulated genes and the number of differentially expressed genes in those two species (Fig. 6A). Moreover, the genes that were differentially expressed among the experimental lines were compared, and the log fold-changes in expression levels were calculated (Tables of Fig. 6B,C).

Among the differentially expressed genes, with high expression in the Lb_SSG_R line, we highlight those genes that encode transporter proteins and that have been described in previous studies. For example, a zinc transporter 3 (LbrM.28.2110), an iron/zinc transporter protein-like protein (LbrM.31.3490), and a putative pteridine transporter (LbrM.06.1250) (Fig. 6B and Supplementary Table S4). Two genes encode histone 4 (LbrM.35.0020 and LbrM.35.0070), one gene encodes a putative heat shock protein 90 (LbrM.29.0780), and one gene encodes a putative 60 S ribosomal protein L28 (LbrM.11.0910), whose function in the SSG-resistant *Leishmania* species is still unknown (Fig. 6B and Supplementary Table S4).

We also observed downregulated expression in the Lb_SSG_R line, including one gene encoding a putative superoxide dismutase (LbrM.32.2860), six genes encoding members of the amastin family (LbrM.08.0680, LbrM.18.0470, LbrM.24.1590, LbrM.20.4290, LbrM.20.4300, and LbrM.20.1080), five genes encoding transporter proteins, three of which representing a putative folate/biopterin transporter (LbrM.10.0380, LbrM.10.0370, and LbrM.34.5090) and two of which encoding a putative pteridine transporter and an ABC transporter (LbrM.10.1460 and LbrM.33.1550, respectively) (Fig. 6B and Supplementary Table S4).

A Gene Ontology enrichment analysis of the genes differentially expressed between *L*. *braziliensis* SSG_S and SSG_R lines (fold-change >2) indicated the enriched terms among the upregulated genes were mainly associated with phosphate ion transport. In contrast, the enriched terms among the genes exhibiting downregulated expression were mainly related to deoxynucleoside catabolism (Fig. 6B).

Although many of the differentially expressed genes in the *L*. *panamensis* lines encode hypothetical proteins with unknown function, some of these genes with significantly upregulated or downregulated expression are mainly associated with the mechanisms underlying antimony resistance that have been described in other *Leishmania* species. Here, *L*. *panamensis* genes were listed along with their corresponding *L*. *braziliensis* ortholog genes that were used for this Gene Ontology enrichment analysis since *L*. *panamensis* genes were not yet integrated in TritrypDB at the time of this analysis.

Of the genes that exhibited upregulated expression in the Lp_SSG_R line, four (LpanUA.27.0220/LbrM.27.0220, LpanUA.10.0410/LbrM.10.0370 LpanUA.14.1530/LbrM.14.1530, and LpanUA.27.1050/LbrM.02.0350) were annotated as encoding a putative vacuolar iron transporter 1, a putative folate/biopterin transporter, a pteridine transporter, and an ABC1 transporter, respectively. Additionally, we observed that LpanUA.27.0140/LbrM.27.0140, encoding a putative WW domain/zinc finger C-x8-C-x5-C-x3-H-type protein, had the highest CNV and was the most differentially expressed gene in the Sb^III^-resistant line (4-fold higher expression in the Sb^III^-resistant line than in the Sb^III^-sensitive line) (Fig. 6C and Supplementary Table S5).

The genes that exhibited downregulated expression in the Sb^III^-resistant line included LpanUA.23.0490/LbrM.23.0490 and LpanUA.23.1530/LbrM.23.0490, which respectively encode trypanothione synthetase and a succinate dehydrogenase-fumarate reductase iron-sulfur protein. The expression levels of these genes were approximately 2-fold lower in the Sb^III^-resistant line than in the Sb^III^-sensitive line (Fig. 6C and Supplementary Table S5).

Finally, the Gene Ontology enrichment analysis of *L*. *panamensis* revealed that among the genes exhibiting upregulated expression, the enriched terms were mainly associated with the regulation of the microtubule cytoskeleton organization, whereas the genes with downregulated expression were mainly enriched with terms associated with the anchoring of microtubules (Fig. 6C).

## Discussion

This study is the first whole genome scale investigation of the impact of Sb^III^ on the genomes and transcriptomes of two of the most important *Leishmania* species in Latin America. We identified significant genomic and transcriptomic differences between the sensitive and resistant lines in both species. The main findings obtained in this study were: (i) Somy changes were observed in both species, being more prominent in the Lb_SSG_R line than in the Lp_SSG_R line. (ii) A high copy number variation affecting 23 genes was observed in the chromosome 27 of Lp_SSG_R line. (iii) The most striking SNPs changes were seen over the long middle section of chromosome 32. (iv) The transcriptional changes were mainly driven by the corresponding genomic copy number changes.

Among the chromosomes that presented somy change, we highlight the chromosomes 11, 13, 23, and 31 in the Lb_SSG_R line and the chromosome 23 in the Lp_SSG_R line due to that similar somy changes were observed in the same chromosomes of the Sb^III^-resistant *L*. *guyanensis*^43^, *L*. *infantum*^27^, *L major*^28^, and *L*. *donovani*^38^ mutants. Although we could not conclude that the observed somy changes were directly responsible for the Sb^III^-resistance of the subgenera/species, we believe that the gene dosage effects caused by chromosome copy number changes are likely critically linked to Sb^III^ resistance phenotype. Despite that the aneuploidy is usually lethal or result in severe abnormalities in some eukaryotes, in others such as *Candida albicans* or *Cryptococcus neoformans*^44^ and in some trypanosomatids (*L*. *major*^45^, *L*. *donovani*^41,45^, *L*. *tropica*^45^, and *T*. *cruzi*^46^), have been identified to be beneficial trait that provides survival advantages. Particularly aneuploidy has been shown to play a key role in environmental adaptation^41^, virulence^47^ and acquisition of Sb^III^ drug resistance^32,34^. Thus, we believe that the gene dosage effects caused by chromosome copy number changes facilitate the acquisition of Sb^III^ resistance phenotype.

A second major genomic change observed in our experimental lines that may be associated with the adaptation of the species to Sb^III^ was related to local CNVs, which were not affected by somy changes. Specifically, the amplified genes in the Lb_SSG_R line and the telomeric amplification of more than 10 fold, involving 23 genes for chromosome 27, in Lp_SSG_R line (Fig. 2) confirmed the results previously reported^43,48,49^. This last finding observed in the Lp_SSG_R line results were interesting, since *Leishmania* subgenus *Viannia* species have never been observed to form episomes because of RNAi mechanisms. Previous studies suggested that *Leishmania* species and other parasites (*P*. *falciparum* and *T*. *cruzi*) use CNVs at specific loci to develop drug resistance^25,28,31,35,43,50,51^, to promote the tissue tropism^40^ and enable adaptations to culture conditions^52^. In this study, we observed that *L*. *braziliensis* and *L*. *panamensis* took advantage of CNVs to modulate the gene dosage in response to Sb^III^, similar to other species such as *L*. *major*^28^, *L*. *tropica*^42^ and *L*. *guyanensis*^43^.

Likewise, our results demonstrated the relationship between aneuploidy and gene CNVs in *L*. *braziliensis*, but not in *L*. *panamensis*. The 92% of genes that presented copy number variation in Lb_SSG_R line, were present in chromosomes with change in the somy, while in Lp_SSG_R line, the genes that presented copy number variation were in chromosomes where the somy was not affected (Fig. 3A,B). These genomic analyses revealed that *L*. *braziliensis* and *L*. *panamensis*, like other *Viannia* species (*L*. *guyanensis)*, use genome structural changes (aneuploidy and/or gene CNVs) as a response to Sb^III^ pressure. However, it remains unclear whether the observed genomic changes are related to Sb^III^ resistance or they are just an adaptive mechanism to overcome external stresses. Resolving this uncertainty will require comprehensive analyses of naturally-occurring antimony-resistant clinical isolates.

Another genomic strategy used by *Leishmania* species to circumvent the effects of drug pressure involves the generation of nucleotide-level variations (SNPs and indels). Some SNPs have been identified as related to the antimony resistance phenotype (e.g., a SNP detected in a gene encoding a protein kinase in *L*. *infantum* or in the *AQP1* gene of *L*. *donovani* and *L*. *guyanensis* strains^27,35,43^). Despite that we did not observe several changes to SNPs level and Indels in the analyzed *L*. *panamensis* and *L*. *braziliensis* lines (Sb^III^-sensitive and -resistant lines), the SNPs on chromosome 32 of *L*. *braziliensis*, which did not lead to changes in the gene dosage and chromosomal somy (Fig. 4), suggest that this species can generate nucleotide-level changes associated with drug resistance without the need for alteration to the genomic structure and gene expression (Table 1). Another interesting finding regarding chromosome 32 was the presence of 32 SNPs in the Sb^III^-sensitive line, but not in the Sb^III^-resistant line. Most of these 32 SNPs were detected in genes encoding hypothetical proteins, but one was in the gene encoding a transmembrane protein (aquaporin) (Table 1), which is reportedly involved in multiple physiological processes in the parasite, such as nutrient absorption and end-product efflux^53^. Although the reason for these genomic changes is unknown and will need to be further investigated, we believe that the behavior observed in our Lb_SSG_R line may be associated to an unconventional recombination event, which can be considered as an analogous mechanism like somy change in a partial chromosome scale.

The pairwise comparison herein reported demonstrates how differences in chromosome numbers and intrachromosomal amplifications or deletions directly affect gene expression in the Lb_SSG_R line (Fig. 5A. Supplementary Fig. S2). However, in the Lp_SSG_R line, we detected extreme gene CNVs on chromosome 27 that were not associated with somy changes (Figs 1 and 2). The increased copy numbers directly resulted in the overexpression of some genes described as molecular targets of Sb^III^ (e.g., a gene encoding the WW domain/zinc finger protein)^54^, as well as genes involved in virulence and in vital biological processes such as parasite growth and survival (elongation factor, alpha- and beta-tubulin)^47^ (Supplementary Fig. S2). Similar synchronized changes between genomic and transcriptomic level were also found critical in other *Leishmania* species and others parasites (e.g., *Plasmodium falciparum)*^55^ that undergoes intrachromosomal amplifications to influence transcript abundance. We believe that our study would have benefited from the inclusion of more than one Sb^III^-resistant mutant line per species. Moreover, we think that additional studies, such as quantitative real-time PCR assays, cloning, and transfections and gene editing, will enable to test the observed overexpression of the genes in the Lp_SSG_R line. These future studies would also clarify the contribution of this gene to the antimony resistance phenotype. Additionally, we believe that the observed drastic changes to chromosome 27 are biologically important, and should be investigated in greater detail (e.g., analyses of infected animals and the effects of resistance or virulence).

Similar to the results of previous studies regarding other parasites, such as *P*. *falciparum*^55^, we observed that our Sb^III^-resistant *L*. *braziliensis* and *L*. *panamensis* lines carried genes with CNVs that did not affect the transcript level (e.g. a gene encoding mannosyl transferase-like protein or encoding putative polyubiquitin present in Lb_SSG_R and Lp_SSG_R lines, respectively) (Supplementary Fig. S3). We also detected varying transcript levels that were not related to differences in gene copy numbers (e.g. a gene encoding 40 S ribosomal protein S15A putative or encoding cysteine peptidase, Clan CA, family C2, putative present in Lb_SSG_R and Lp_SSG_R lines, respectively) (Supplementary Fig. S3). Interestingly, some of these genes were shown to be associated with the resistance of *L*. *donovani*, *L*. *tarentolae*, *and L*. *major* to antimonials^38,56^. Increases in gene expression levels without gene amplifications or vice versa may be explained by a lack of transcriptional regulation, which has been described for *Leishmania* species. Various studies have determined that this behavior is probably due to co-amplification of another nearby gene, or an increase in RNA stability, which promotes expression, or a mutation in a promoter-like element of an upstream gene^55,57,58^. These explanations are also likely applicable to our results. Finally, the pairwise comparison of genes differentially expressed in the SSG_S and SSG_R lines suggested that gene expression confers a degree of plasticity to these species under Sb^III^ pressure. For example, these lines may undergo changes to the expression of genes mediating antimony resistance as well as genes related to virulence and vital biological processes, including parasite growth and survival (Fig. 6A–C).

One limitation of our study was the use of the promastigote stage rather than intracellular amastigote stage, which has been considered as the gold standard for *in vitro Leishmania* drug discovery research and evaluation of resistance^59,60^, and despite that experimental evidence (using mainly microarrays) have suggested that the transcriptomic behavior between amastigote and promastigote stages is different^61,62^. We consider that the molecular changes observed can be used for future WGS studies using amastigotes stage. Our findings represent the baseline for future studies that conduct genomic and transcriptomic analyses on amastigotes and depict the molecular features associated to the Sb^III^ response. Likewise, and taken into account that in *Leishmania* parasites, the expression of individual genes is regulated post-transcriptionally, due to the absence of promoter-mediated regulation of transcription initiation of nuclear genes^42,63^, which can result in a variable correlation between gene and protein expression level^64^. We believe that future analysis should consider how the transcriptomic findings observed in this study could have a relationship with the final protein dosage. We also recommend conducting comprehensive and robust proteomic studies to unravel the true proteins associated in response to drug pressure.

In conclusion, this whole genome scale DNA-seq and RNA-seq study highlighted the importance of gene dosage effects of genomic and transcriptional levels as the coping mechanisms against the antimony exploited by *L*. *braziliensis* and *L*. *panamensis*. These two species still remain to be enigmatic parasites which can cause disfiguring mucocutaneous leishmaniasis. Our data would serve as a first step towards the better understanding of the genomic and transcriptomic changes caused under the SSG stress *in vitro* and should also provide the basis for future studies examining the applicability and commonality of the genomic and transcriptomic changes observed in the study to the parasites encountered in the clinical setting (i.e., strains with natural resistance). It is our hope that the further sequencing and molecular analyses on experimental and clinical SGG resistance will be able to contribute to the identification of new therapeutic targets in the near future.

## Methods

### Culture conditions and development of drug-resistant *Leishmania braziliensis* and *Leishmania panamensis* promastigotes

Promastigotes of *L*. *braziliensis* (MHOM/BR75/M2904) and *L*. *panamensis* (MHOM/COL/81/L13) sensitive to Sb^III^ (SSG_S) and resistant to Sb^III^ (SSG_R) were axenically maintained in RPMI 1640 medium (Sigma-aldrich) supplemented with 10% (v/v) heat inactivated fetal bovine serum (Invitrogen) and culture at 26 °C with 5% CO_2_.

The Sb^III^-resistant population, [*L*. *braziliensis* (Lb_SSG_R) and *L*. *panamensis* (Lp_SSG_R)] were obtained from wild-type sensitive *L*. *braziliensis* (Lb_SSG_S) and *L*. *panamensis* (Lp_SSG_S) via the continuous stepwise increase in drug pressure with Sb^III^, as described by Liarte and Murta^65^. For each species, selection of resistant parasites was initiated in quadruplicates. Briefly, 10^6^ logarithmic-phase promastigotes were incubated with 1.5 μg/ml Sb^III^ (*L*. *braziliensis)* or 1.8 μg/ml Sb^III^, (*L*. *panamensis)*. This concentration corresponds to the effective concentration that inhibits growth by 50% (EC_50_). The drug concentration was increased in a stepwise manner only when the drug-exposed parasites had a growth rate similar to that of the parental parasites. Selection rounds were performed successively at 2-fold increase with 1.5, 3, 6, 12, 24 and 48 μg/ml Sb^III^ for *L*. *braziliensis* and 1.8, 3.6, 7.2, 14.4, 28.8 and 57.6 μg/ml Sb^III^ for *L*. *panamensis*. This increment was continued until the maximum concentration allowing parasite growth was reached. After this period, the SSG_R lines were maintained for 3 weeks at the final drug concentration. To verify that the observed drug resistance phenotypes were stable, we cultivated the Sb^III^-resistant lines for 4 weeks in the absence of Sb^III^. The Sb^III^-sensitive *Leishmania* species were cultured in parallel, but without any drug pressure. At the end of this period, the susceptibility of the sensitive and resistant lines to Sb^III^ was determined by calculating the EC_50_ in an MTT [3-(4,5-dimethylthiazol-2-yl)-2,5-diphenyltetrazolium bromide] colorimetric assay, as previously described^66^. The corresponding absorbance values were obtained and the EC_50_ was calculated using the Graph Pad Prism 5.0. The assays were performed three times in triplicate. Differences in the data were considered significant when the resistance index was ≥10-fold different between the Sb^III^-resistant and -sensitive lines.

### Isolation of RNA and DNA

Approximately 1 × 10^6^ promastigotes (sensitive and resistant to Sb^III^) in the late logarithmic growth phase were cultured and harvested by centrifugation. The resulting pellets were divided in two equal parts, for DNA and RNA extraction.

Total RNA was extracted from four independent replicates (two technical and two biological replicates) of each Sb^III^-resistant and -sensitive line, each originating from a separate culture. The RNA was extracted with the RNeasy Mini Kit (Qiagen, USA), and the DNA was extracted from one replicate of each Sb^III^-resistant and -sensitive line with the High Pure PCR Template Preparation Kit (Roche Life Science). The RNA and DNA concentrations were determined with the NanoDrop ND-1000 spectrophotometer (Thermo Scientific, USA). The RNA quality and integrity were assessed with the 2100 Bioanalyzer system (Agilent Technologies, Santa Clara, CA, USA) according to the manufacturer’s instructions. The DNA quality and integrity were determined by 1% agarose gel electrophoresis. All samples had an A_260_/A_280_ ratio greater than 2.0.

### Genome and transcriptome sequencing

The mRNA, cDNA libraries and the extracted whole genome DNA were prepared and sequenced with the HiSeq X-Ten system (Illumina) by Novogene Bioinformatics Technology Co., Ltd, Beijing, China. Paired reads of 75 nucleotides were obtained for the mRNA libraries, whereas 2 × 100 bp reads length were obtained for the cDNA libraries. Sequence quality metrics were assessed with FastQC (Illumina platform, PE 150, Q30 ≥80%; 250–300 bp insert cDNA library). Additionally, the 20 million raw reads/sample rRNA depleted was completed according to the poly(A) magnetic beads capture protocol, with the Strand-specific TruSeq RNA-seq Library Prep kit (Illumina), which was also used to prepare libraries.

To whole genome DNA, the mate-paired libraries constructed by end repair (350-bp insert size) were subjected to paired-end sequencing (2 × 150-bp read length).

### Genome data analysis

Paired-end Illumina reads were mapped to the reference MHOM/BR75/M2904 *L*. *braziliensis* genome sequence and the UA946 *L*. *panamensis* genome sequence assembly with the SMALT program (version 0.7.4) (www.sanger.ac.uk/resources/software/smalt/). The mapping involved the following parameters: exhaustive search option (−x and −y 0.8); a reference hash index of 13 bases; and a sliding step of 3. An identity threshold of y = 0.8 prevented the mapping of non-*Leishmania* reads to the reference sequences because SMALT can trim reads before mapping them to the reference sequence. The read file merging, sorting, and elimination of PCR duplicates were implemented with SAMtools (version 0.1.18) and Picard (version 1.85)^35^.

For the chromosomal somy estimation, the median read depth of each chromosome was initially calculated (d_i_). All positions with a read depth >1 standard deviation away from this initial median were then removed, and the d_i_ was recalculated. This approach removed depth outliers due to assembly errors, local CNVs, or spurious high-coverage regions influencing the final median. Subsequently, the median depth of the 35 chromosomes (d_m_) for *L*. *braziliensis and L*. *panamensis* was calculated, and the somy (*s*-value) of each chromosome was obtained with the following formula: *s* = 3 × d_i_/d_m_ (for *L*. *braziliensis*) and *s* = 2 × d_i_/d_m_ (for *L*. *panamensis*)^37^. Among our samples, the shorter chromosomes did not tend to deviate from the expected somy^41^. The somy values calculated from sequencing data are averages across the potentially variable somy of these cells. For this reason, somy values may be noninteger values, representing the mean value of a mixed population. The range of monosomy, disomy, trisomy, tetrasomy, and pentasomy was then used to define the full cell-normalized chromosome depth or somy (S) as S < 1.5, 1.5 ≤ S < 2.5, 2.5 ≤ S < 3.5, 3.5 ≤ S < 4.5, and 4.5 ≤ S < 5.5, respectively, as previously described^41^. Because the depth of samples was sufficient, we did not test other normalization factors, such as various percentile depths or a statistically weighted normalization factor. To evaluate the CNVs at the gene level, we defined an average haploid depth per gene without the somy effect as $\bar{d}\,$_*HG*_, and defined the full cell depth with the somy effect as $\bar{d}\,$_*FG*_. Their relationship was defined as $\bar{d}\,$_*FG*_ = *S*$\cdot \bar{d}$_*HG*_.

Two criteria were used to evaluate whether differences in the gene or chromosome copy number between the Sb^III^-sensitive and -resistant lines were biologically and statistically significant. The first requirement was that the absolute difference in the gene/chromosome copy number between the Sb^III^-sensitive and -resistant lines should be at least 0.5^41^. Second, the false discovery rate (FDR) adjusted p-value (Student’s *t*-test and Benjamini–Hochberg correction) had to be lower than 0.05. Heatmaps were created using the Heatmap3 package in R^67^. All gene IDs reported herein for *L*. *panamensis* were based on the orthologous genes in the *L*. *braziliensis* genome.

To detect the single nucleotide polymorphisms and insertions/deletions, the reads were aligned to the reference MHOM/BR75/M2904 *L*. *braziliensis* genome sequence or the UA946 *L*. *panamensis* genome sequence assembly, using the Smalt program (version 0.7.4) (http://www.sanger.ac.uk/science/tools/smalt-0). The Picard program (version 1.85) (http://broadinstitute.github.io/picard/) was used for merging and sorting bam files and marking duplicated reads, as previously described^41^. The SNPs and insertions/deletions (indels) shorter than 15 bp were called with the population-based Unified Genotyper method in the Genome Analysis Toolkit (GATK) (version 3.4; https://software.broadinstitute.org/gatk/). DNA regions with more than three SNPs within 10 bases of each other were marked as SNP clusters and were maintained for subsequent analyses. Low-quality SNPs were filtered by GATK Variant Filtration with QD < 2.0 || MQ < 40 || FS > 60.0 || ReadPosRankSum < −8.0. To avoid false negatives, the SNP quality cut-off was set to 300. All candidate SNPs were visually inspected in the Integrative Genomic Viewer (IGV_2_3_47)^41^ and SAMtools to avoid false positives. The SnpEff program (version v4.1)^41^ was used to classify all SNPs and indels based on their functional impact. The SNPs and indels were compiled in a population genetic variation vcf file. From this vcf file, alternative allele and depth information was extracted for further analysis.

The SNPs and small indels were considered significantly different between the Sb^III^-sensitive and -resistant lines when the allele shift difference was at least 0.25 for *L*. *panamensis* and at least 0.33 for *L*. *braziliensis*^68^ with a Mann–Whitney U test p-value < 0.05. Allele shifts larger than 0.80 were considered homozygous variants.

### Transcriptome data analysis

Transcript abundance was quantified by assessing read depth, as previously described^25,35^ and multiply mapped reads were kept in the analysis to quantify repetitive genes. For each chromosome, the average transcript depth was used to compute an RNA-based relative somy value, (i.e., RNA-S). The correlation between DNA and RNA depth, namely between S and RNA-S, was calculated and visualized with SciPy^69^. For differential expression analysis, STAR (2.5.2) was used for mapping and read counting per gene with default parameters where multiply mapped read were marked and ignored. DEseq2 (version 1.18.1) was then used to normalize the read counts and evaluate the statistical significance of differentially expressed genes. Here the following criteria were used: a fold-change cut off ≥2 and a Benjamini–Hochberg adjusted p-value < 0.05. The percentage of differentially expressed genes per chromosome was defined as follows: (number of differentially expressed genes per chromosome)/(number of total genes per chromosome) × 100.

Gene Ontology enrichment analyses were performed using Tritrypdb tools (http://tritrypdb.org) with Fisher exact test used to maintain the FDR below 0.05. The GO terms were submitted to REVIGO^70^.

Finally, Venn diagram was constructed using a program provided by the Bioinformatics and Evolutionary Genomics group of the University of Gent and the VIB institute (http://bioinformatics.psb.ugent.be/webtools/Venn/).

## Supplementary information

Supplementary material

Supplementary dataset
