# Supplementary material for: Major changes in chromosomal somy, gene expression and gene dosage driven by SbIII in Leishmania braziliensis and Leishmania panamensis
Source: Sci Rep. 2019 Jul 1;9:9485. doi: 10.1038/s41598-019-45538-9 (PMC6603004; doi:10.1038/s41598-019-45538-9)
Supplement: Supplementary file 1 — Supplementary material [file 41598_2019_45538_MOESM1_ESM.docx]

***Supplementary Material***

**Major changes in chromosomal somy, gene expression and gene dosage driven by SbIII in *Leishmania braziliensis* and *Leishmania panamensis***

Luz H. Patino^1^, Hideo Imamura^2^, Lissa Cruz-Saavedra^1^, Paula Pavia^3^, Carlos Muskus^4^, Claudia Méndez^5^, Jean Claude Dujardin^2,6^, Juan David Ramírez^1^*

^1^ Grupo de Investigaciones Microbiológicas-UR (GIMUR), Programa de Biología, Facultad de Ciencias Naturales y Matemáticas, Universidad del Rosario, Bogotá, Colombia.

^2^ Molecular Parasitology Unit, Department of Biomedical Sciences, Institute of Tropical Medicine, Antwerp, Belgium.

^3^ Unidad de Investigación Científica, Subdirección de Docencia e Investigación, Hospital Militar Central, Bogotá, Colombia

^4^ Programa de Estudio y Control de Enfermedades Tropicales (PECET), Facultad de Medicina, Universidad de Antioquia, Medellín, Colombia.

^5^ Dirección de Sanidad Militar, Ejercito Nacional de Colombia, Bogotá, Colombia.

^6^ Department of Biomedical Sciences, University of Antwerp, Belgium

*Corresponding author Address: Carrera 24 # 63C - 69, Bogotá, Colombia

Telephone number: +57-1-297 0200 Ext: 4033

E-mail address: juand.ramirez@urosario.edu.co

**Supplementary Material**

**Summary**

The supplementary material includes 3 figures and 5 tables.

**Supplementary information legends**

**Supplementary Figure S1. Neighbor-joining dendrogram of *L. braziliensis* and *L. panamensis*.** The scale of the networks is the number of base substitutions per site. **A.** *L. braziliensis* **B.** *L. panamensis* where W and I represent SSG sensitive and resistant lines, and D represent genomic data and suffix numbers represent RNA sample IDs.

**Supplementary Figure S2. Relationship between the gene copy number variation and the gene expression, evaluated in SSG_S and SSG_R lines of *L. braziliensis* and *L. panamensis*.** Lists of the genes that presented CNV and alteration in the gene expression (DE more than 1.5-fold) in **A.** *L. braziliensis* and **B.** *L. panamensis*. The negative values indicates a higher expression in the resistant than that in the sensitive line.

**Supplementary Figure S3.** Lists of the genes that presented CNV between SSG_S and SSG_R (difference >1.0) without the gene expression change (DE more than 1.5-fold) in **A.** *L. braziliensis* and **C.** *L.panamensis*. Lists of the genes that did not present CNV between SSG_S and SSG_R (difference >1.0) but presented the gene expression change (DE more than 1.5-fold) in **B.** *L. braziliensis* and **D.** *L. panamensis* .

**Supplementary Table S1**. List of the genes located in the first 50,000 bp of chromosome 27 in the Lp_SSG_R line with signiticant difference compared to Lp_SSG_S line

**Supplementary Table S2**:

**A.** List of the genes that presented CNV between the Lb_SSG_S and Lb_SSG_R lines in chromosomes that changed their S values

**B.** List of genes that presented CNV between the Lb_SSG_S and Lb_SSG_R lines in chromosomes that did not changed their S values (Excel File)

**Supplementary Table S3**. List of heterozygous SNPs held an difference in the allele frequency (greater than 0.33) between the Lb_SSG_S and Lb_SSG_R lines. (Excel File)

**Supplementary Table S4**. List of differentially expressed genes between the Lb_SSG_S and Lb_SSG_R lines with a fold-change ≥ 2. (Excel File)

**Supplementary Table S5**. List of differentially expressed genes between the Lp_SSG_S and Lp_SSG_R lines with a fold-change ≥ 2. (Excel File)

**Supplementary Fig. S1**

**Supplementary Fig. S2**

**Supplementary Fig. S3**

**Supplementary Table S1**
